# Characteristics of older patients in the largest French psychiatric emergency centre

**DOI:** 10.3389/fpsyt.2023.1298497

**Published:** 2023-12-12

**Authors:** Alexandra Pham-Scottez, Thierry Gallarda, Mathilde Calvez, Jérôme Silva, David Barruel, Valérie Dauriac-Le Masson, Justine Lahaye, Florence Perquier, Marie Sarazin, Raphaël Gourevitch

**Affiliations:** ^1^Centre Psychiatrique d’Orientation et d’Accueil (CPOA), GHU Paris Psychiatrie & Neurosciences, Hôpital Sainte Anne, Paris, France; ^2^Centre d’Evaluation des Troubles Psychiques et du Vieillissement (CETPV), GHU Paris Psychiatrie & Neurosciences, Hôpital Sainte Anne, Paris, France; ^3^Hôpital Lariboisière, AP-HP, Paris, France; ^4^Département d’Information Médicale (DIM), GHU Paris Psychiatrie & Neurosciences, Hôpital Sainte Anne, Paris, France; ^5^Cellule Epidémiologie, GHU Paris Psychiatrie & Neurosciences, Hôpital Sainte Anne, Paris, France; ^6^Centre for Addiction and Mental Health, Cundill Centre for Depression and Mental Health, Toronto, ON, Canada; ^7^Department of Neurology of Memory and Language, GHU Paris Psychiatrie & Neurosciences, Hôpital Sainte Anne, Paris, France; ^8^Université Paris Cité, Paris, France; ^9^Université Paris-Saclay, BioMaps, Service Hospitalier Frédéric Joliot CEA, CNRS, Inserm, Orsay, France

**Keywords:** older people, older patients, psychiatric emergency department, psychiatric emergencies, gerontopsychiatry

## Abstract

Despite an increasing number of adults older than 60 years with psychiatric disorders, there are few studies on older patients in psychiatric emergencies and no European data. We aimed to describe the population of patients aged 60 years and older who presented to the main French psychiatric emergency centre and identify predictors of psychiatric hospitalization. This monocentric study included 300 consecutive patients aged 60 years and older. Patients presenting because of psychiatric emergencies were frequently female and lived autonomously. More than 40% had a history of at least one psychiatric hospitalization and 44% had consulted a psychiatrist in the previous 6 months. The most common reasons for consultation were depression, anxiety, sleep disorders and suicidal thoughts. Psychiatric disorders were mainly mood disorders; neurotic, stress-related and somatoform disorders; and schizophrenic, schizotypal and delusional disorders. Only 10% had a diagnosis of organic mental disorders. Overall, 39% of the patients were admitted to the psychiatric hospital. Factors predicting hospitalization were a history of psychiatric hospitalization, suicidal thoughts and a diagnosis of a mood disorder or schizophrenia/schizotypal/delusional disorder. In conclusion, among people aged 60 years and older who consulted for psychiatric emergencies, 39% had to be hospitalized in psychiatry and only psychiatric factors influenced the decision to hospitalize. Our study highlights the need for further studies of older people in psychiatric emergencies in Europe, to anticipate the needs of this specific population and adapt multidisciplinary mental health care.

## 1 Introduction

Population aging, in France as in all Western countries, has led to a significant increase in the number of adults older than 60 years who have psychiatric disorders. According to the World Health Organization (WHO), psychiatric disorders are one of the leading causes of morbidity and premature mortality in older people and constitute three of the five sources of disability related to ageing (1). Moreover, the proportion of older people will continue to grow (2). As of 2022, 21.6% of Parisians were older than 60 years, and by 2030 this figure will increase to 24%. With this demographic explosion, the psychiatry of older people is becoming a real public health issue. We need rigorous and reliable data to better understand the current and future needs of this population (3) in order to anticipate and adapt a multidisciplinary healthcare offer.

Studies of the use of psychiatric emergency services (PES) by older people are relatively few and were mainly by North American teams. The publications are listed in Table 1. The first study (4) compared 49 patients over age 65 years, with 49 patients between 40 and 65 years consulting for PES in Philadelphia. As compared with middle-aged adults, older adults were more often new psychiatric patients, more often came with a close accompanying person, and had more organic brain disorders and fewer alcohol- and schizophrenia-related issues. The same year, patients older than 60 years who consulted for PES in Philadelphia were assessed (5). The most frequent diagnoses were organic brain syndrome (45.5%), schizophrenia (25.6%) and depression (13.9%). A similar study found that patients older than 65 years represented a mere 2.9% of those consulting for PES in Cincinnati, Ohio (6) and they were hospitalized more frequently than were young people. Another US study (7) included 118 older patients consulting for PES in Dallas, Texas; half had a psychiatric history. The main diagnoses were organic brain disorders, mood disorders and abuse of drugs or misuse of medications. In New Jersey, patients over 60 years accounted for 23.8% of emergency psychiatric visits (8); the most common diagnoses were dementia, affective disorders, schizophrenia and alcohol disorders. A recent US study (9) showed an increase in the number and proportion (6.2%) of consultations by people older than 65 years in an emergency psychiatric centre in Hawaii during 2007–2011. The most common diagnosis was dementia (14%).

**Table 1:** Summary of the literature.

Author	Year of publication	Country	Centre	Age, years	Patients	Main conclusions
Waxman (4)	1982	United States	Monocentric	65	49 (>65 years) compared with 49 (40–64 years)	More first contact with psychiatry More accompanied with family and friends Less substance abuse and schizophrenia More organic brain syndrom
Simson (5)	1982	United States	Bicentric	60	180 from a psychiatric emergency service and 170 from a geriatric emergency service	Comparison between the two services
Thienhaus (6)	1988	United States	Monocentric	65	118 (>65 years) compared with 118 (<65 years)	More hospitalizations Dementia increases the likehood of hospitalization only if comorbid with another psychiatric diagnosis No effect of medical comorbidity
Puryear (7)	1991	United States	Monocentric	65	118 (254 charts [>65 years], half randomly studied of 8,561 visits)	Half with a history of psychiatric illness One third with organic brain disease 27 mis-prescriptions or misuse of medications Not real emergencies, often complaints for more than 6 months
Coyne (8)	1993	United States	Monocentric	60	105 23.8% of adult visits	27% dementia 27% affective disorders 16% schizophrenia 12% psychosis 7% alcohol abuse
Lu (9)	2017	United States (Hawaï)	Monocentric	65	1,370 (of 22,124 visits)	Increase of numbers and proportions (>65 years) between 2007 and 2011 Increase of violent behaviors between 2007 and 2011 Longer length of stay (>65 years) Longer length of stay (>80), only in case of hospitalization
Chaput (10)	2011	Canada	Four centres	65	1,349 (>65 years) 8.7% of the patients 7.2% of the visits	The media nage increases over time (2002 to 2004) More often women (62%), widowed (28%), accompanied (42%), with depression or anxiety (39%) Illness more often reported as an important stressor (29%) Less violence at arrival (10%) More hospitalizations (40%)
Perez (11)	1986	Canada	Monocentric	60	58 (>60 years) compared with 931 (17–59 years)	More widowed or married than single Chief complaint for a longer time before arrival More with affective disorders, more psychological factors affecting physical condition, and less with personality disorders
Niizato (12)	2003	Japan	Four centres	60	584 (>60 years) of 7,971 over a 4-year period	Increase of the annual number of patients (>60) 99% of mandatory admissions Distribution of psychiatric diagnoses is very different
Sawayama (13)	2009	Japan	monocentric	65	14 (>65 years) compared with 277 (>65 years) police-call cases	More organic mental disorders, mood disorders, dementia, disturbed consciousness More suicide attempts in case of mood disorder No excitation Less history of visiting a psychiatrist Less history of hospitalization

The largest published study is a longitudinal observational study (10) of four psychiatric emergency centres in Quebec, Canada, between 1990 and 2004; 8.7% of patients were older than 65 years. The older-age population included 62% females, and 28% were widowed; 42% were accompanied by a close relative, and 29% reported a somatic illness as a significant stressor. Mood disorders were the most common diagnoses. Those older than 65 years seemed less comorbid than those less than 65 years and less frequently presented violent behavior. Finally, psychiatric hospitalization more frequently resulted from consultations for people older than 65 years than those less than 65 years.

Another Canadian study (11) included 989 patients who consulted for PES; 5.9% were older than 60 years old. As compared with people younger than 60 years, those older than 60 had a longer duration of their main complaint before being referred to the emergency department by their general practitioner and a higher prevalence of affective disorders and psychological factors affecting their physical condition. Also, older people were more frequently admitted to inpatient psychiatric services after consultation.

The only two Asian studies are from Japan. The first included 7,971 patients seen in an emergency psychiatric centre in Tokyo from 1998 to 2001 (12). Patients older than 60 years represented 7.3% of the patients (this proportion increased each year from 1998 to 2001). The second (13) compared patients older than 65 years (4.8% of the 291 patients) to patients younger than 65 years: older patients were frequently male and had dementia and mood disorders. They less frequently had schizophrenia and a history of psychiatric visits or psychiatric hospitalization than younger patients.

To date, we have not found any studies on older patients presenting to European psychiatric emergency departments. We aimed to describe (in terms of socio-demographic and clinical characteristics, both diagnostic and orientation) the population of patients aged 60 years and older who consulted in the largest psychiatric emergency centre in France and to identify predictors of psychiatric hospitalization.

## 2 Methods

### 2.1 Study site

The *Centre Psychiatrique d’Orientation et d’Accueil* (CPOA), located in Sainte-Anne Hospital, Groupe Hospitalier Universitaire Paris Psychiatrie et Neurosciences, Paris, is the largest psychiatric emergency centre in France (approximately 10,000 consultations per year). The CPOA mainly receives people in psychiatric emergencies but also patients who want to see a psychiatrist without an appointment, 24 h a day, 7 days a week, from Paris, the suburbs and the Ile-de-France region.

### 2.2 Patients

This monocentric observational study included all consecutive patients who consulted the CPOA and met the inclusion criteria: age 60 years and older and signing the informed consent form after having received oral and written information. We excluded patients who had poor comprehension and/or did not speak French, were under full guardianship, were already in compulsory care before their consultation at the CPOA, refused to participate in the study, or were already included in this study (during a previous consultation at the CPOA). Throughout the study, we kept a register of non-inclusions indicating the age, sex and reason for non-inclusion.

### 2.3 Assessments

We collected the following information: sociodemographic data (age, sex, marital status, place of residence, occupation etc.), origin of the request for a CPOA consultation, psychiatric history (history of psychiatric hospitalization, psychiatric consultation less than 6 months, psychotropic treatment less than 1 month, number of psychotropic treatments), level of autonomy (assessed by the investigator on a four-point scale: autonomous/limited assistance/regular assistance/constant assistance, with scoring guidelines), Mini Mental Score Examination (MMSE) score, symptoms and reasons for consultation (one or two, from a list of 21 items), psychiatric diagnoses (according to the International Classification of Diseases, 10^th^ Revision [ICD-10] (14)), and orientation (inpatient or outpatient). A psychiatrist investigator of the study collected data for each patient and then entered the data into an anonymous database.

### 2.4 Ethics of the study

This study was conducted in accordance with the legal provisions governing clinical research in France. The study was approved by the *Comité de Protection des Personnes EST-1* on 27 June 2018. Each patient enrolled in the study signed an informed consent form after receiving oral and written information about the study and its objectives. The anonymous database of the study was declared to the *Commission Nationale Informatique et Libertés.*

### 2.5 Statistical analyses

The characteristics of the population aged 60 years and over are described by proportions for categorical variables and means (+/− standard deviation) for quantitative variables. The associations between the characteristics of patients and their orientation (outpatient or psychiatric hospitalization) after consultation were tested by chi-squared test or Fisher’s exact test for categorical variables and for quantitative variables, by Student *t* test, ANOVA or non-parametric tests (Wilcoxon, Mann–Whitney) in the case of a non-normal distribution.

The probability of being admitted to a psychiatric hospital was then modeled with a logistic regression model. The variables were selected on the basis of their clinical relevance and after verifying their correlation with the likelihood of being admitted to a psychiatric hospital. After a series of bivariate and multivariate evaluations, the most parsimonious logit model was selected for ease of interpretation. The model validity assumptions were checked and we performed several model quality tests.

## 3 Results

### 3.1 Patients not included in the study

Six patients were not included because the investigator forgot or was otherwise busy, two had already been included during their previous consultation at the CPOA, and 11 were unable to consent or participate in the study (due to excessive agitation or sedation). Other patients had at least one exclusion criterion: 74 patients refused to participate in the study, 22 were not fluent in French, 28 were under protective measures, and six were already under compulsory care. Finally, 300 patients were included from 06 September 2018 to 01 November 2019: 182 were outpatients and 118 were admitted to psychiatric hospitals (inpatients).

### 3.2 Description of the whole study sample

The sociodemographic characteristics, psychiatric history, psychotropic treatments and autonomy of the 300 patients are in Table 2. Supplementary Figure S1 shows the age of patients by sex. The distribution of the number of psychotropic treatments per patient is in Supplementary Figure S2.

**Table 2:** Sociodemographic variables, psychiatric history, psychotropic treatments, and autonomy.

Variables	Total sample *N* = 300	Outpatients *N* = 182	Psychiatric inpatients *N* = 118	value of *p* Fisher test (unless indicated)
Age				
Years	69.2 (+/−7.0)	69.4 (+/−7.1)	68.9 (+/−7)	0.578 (t-test)
Sex *N* (%)				
Males Females	123 (41%) 177 (59%)	81 (44.5%) 101 (55.5%)	42 (35.6%) 76 (64.4%)	0.158
Marital status				
Couple/married Single Divorced/separated Widowed	110 (36.7%) 65 (21.7%) 73 (24.3%) 52 (17.3%)	71 (39%) 35 (19.2%) 46 (25.3%) 30 (16.5%)	39 (33.1%) 30 (25.4%) 27 (22.9%) 22 (18.6%)	0.508
Place of residence				
At home Institution Without a home or emergency accommodation	268 (89.3%) 17 (5.7%) 15 (5%)	164 (90.1%) 9 (4.9%) 9 (4.9%)	104 (88.1%) 8 (6.8%) 6 (5.1%)	0.767
Professional activity				
Employed Unemployed Retired Inactive	43 (14.3%) 12 (4%) 220 (73.3%) 25 (8.3%)	28 (15.4%) 9 (4.9%) 130 (71.4%) 15 (8.2.%)	15 (12.7%) 3 (2.5%) 90 (76.3%) 10 (8.5%)	0.688
Psychiatric history				
History of psychiatric hospitalization	125 (41.7%)	57 (31.3%)	68 (57.6%)	0.00001
Psychiatric consultation in the last 6 months	132 (44%)	71 (39%)	61 (51.7%)	0.041
Psychotropic treatments				
Psychotropic treatment in the previous month	224 (74.7%)	130 (71.4%)	94 (79.7%)	0.143
Number of psychotropic treatments (among patients receiving at least one)	2.2 (+/−1.2)	2.1 (+ /−1.2)	2.4 (+/−1.2)	0.039 (*t*-test)
Autonomy				
Independent Requires assistance No information	225 (75%) 67 (22.3%) 8 (2.67%)	145 (79.7%) 33 (18.1%) 4 (2.2%)	80 (67.8%) 34 (28.8%) 4 (3.4%)	0.078

Data are mean (+/− SD) unless otherwise indicated.

For 115 patients (38.3%), the patients themselves were the source of the CPOA consultation, and for 51 (17%), it was a friend or family member. Seven patients (2.3%) were referred by a public psychiatric consultation centre, 20 (6.7%) by a private psychiatrist, 4 (1.3%) by another specialist and 48 (16%) by their general practitioner. The remaining patients were referred by an emergency service (*n* = 17, 5.7%), a hospital service (*n* = 18, 6%), long-term care home staff (*n* = 22.7%), the police (*n* = 2, 0.7%), the fire department (*n* = 1, 0.3%) and other requests (*n* = 9, 3%).

Table 3 summarises the origin of the consultation request, symptoms and reasons for consultation, ICD-10 psychiatric diagnoses and MMSE score. The mean MMSE score for the whole sample was 24.7 (+/−5.4) (range 2 to 30).

**Table 3:** Origin of request for consultation, symptoms and reasons for consultation, ICD-10 psychiatric diagnoses and MMSE score.

Variables	Total sample *N* = 300	Outpatients *N* = 182	Psychiatric inpatients *N* = 118	value of *p* Fisher test (unless indicated)
Origin of request for consultation				
Patient/family or friend Professional	166 (55.3%) 134 (44.7%)	108 (59.3%) 74 (40.7%)	58 (49.2%) 60 (50.8%)	0.1063
Symptoms and reasons for consultation				
Anxiety	126 (42%)	81 (44.5%)	45 (38.1%)	0.33093
Sleep disorders	88 (29.3%)	55 (30.2%)	33 (28%)	0.77257
Eating disorders	26 (8.7%)	14 (7.7%)	12 (10.2%)	0.59271
Somatic complaints	20 (6.7%)	16 (8.8%)	4 (3.4%)	0.09554
Suicidal beaviour, self-injurious behaviors	13 (4.3%)	5 (2.7%)	8 (6.8%)	0.16592
Depressive thoughts	159 (53%)	91 (50%)	68 (57.6%)	0.24017
Suicidal ideation	69 (23%)	28 (15.4%)	41 (34.7%)	0.00018
Heteroagressive behavior	12 (4%)	8 (4%)	4 (3.4%)	0.76987
Restlessness, excitement	24 (8%)	13 (7.1%)	11 (9.3%)	0.64423
Delusions, hallucinations	46 (15.3%)	15 (8.2%)	31 (26.3%)	0.00005
Seclusion, withdrawal, odd behavior	7 (2.3%)	1 (0.5%)	6 (5.1%)	0.01613
Delirium	3 (1%)	2 (1.1%)	1 (0.8%)	1
Wandering, fugue, pathological travel	6 (2%)	2 (1.1%)	4 (3.4%)	0.21601
Silence	6 (2%)	3 (1.6%)	3 (2.5%)	0.68293
Request related to alcohol	12 (4%)	9 (4.9%)	3 (2.5%)	0.3767
Request related to drugs	0 (0%)	0 (0%)	0 (0%)	1
Request for information	2 (0.7%)	2 (1.1%)	0 (0%)	0.52116
Administrative request	2 (0.7%)	3 (1.6%)	0 (0%)	0.28174
Social request (emergency accommodation, social support etc.)	3 (1%)	5 (2.7%)	0 (0%)	0.16065
Other	5 (1.7%)	12 (6.6%)	1 (0.8%)	0.01847
ICD-10 psychiatric diagnoses				
F00-F09 organic, including symptomatic, mental disorders	31 (10.3%)	22 (12.1%)	9 (7.6%)	0.29566
F10-19 mental and behavioral disorders due to psychoactive substance use	19 (6.3%)	12 (6.6%)	7 (5.9%)	1
F20-F29 schizophrenia, schizotypal and delusional disorders	33 (11%)	10 (5.5%)	23 (19.5%)	0.00032
F30-F39 mood (affective) disorders	183 (61%)	101 (55.5%)	82 (69.5%)	0.02106
F40-F48 neurotic, stress-related and somatoform disorders	64 (21.3%)	52 (28.6%)	12 (10.2%)	0.00026
F50-F59 behavioral syndromes associated with physiological disturbances and physical factors	4 (1.3%)	4 (2.2%)	0 (0%)	0.15689
F60-F69 disorders of adult personality and behavior	11 (3.7%)	8 (4.4%)	3 (2.5%)	0.53649
MMSE score				
Mean (+/− SD)	24.7 (+/−5.4)	24.9 (+/−5.6)	24.5 (+/−5)	0.5248 (t-test)

ICD-10, International classification of diseases, 10th revision; MMSE, mini mental score examination.

The outpatient orientations after the CPOA consultation were as follows: psychiatric outpatient follow-up, non-psychiatric outpatient follow-up (including geriatric consultations), both psychiatric and non-psychiatric outpatient follow-up, and post-emergency consultation at the CPOA. For seven patients (2.3%), no medical or psychiatric follow-up was recommended. We referred 10 patients (3.3%) to a medical emergency department. A total of 118 patients (39.3%) were admitted to a public psychiatric hospital. Two patients returned home while waiting for admission to a private psychiatric hospital (non-emergency).

### 3.3 Bivariate and multivariate analyses

Comparisons between inpatients and outpatients are in Tables 2, 3. For these bivariate analyses, the origin of the consultation request was transformed into a binary variable: request from the patient/family/close friends or from professionals (public psychiatric consultation centre/private practice psychiatrist/other professional/general practitioner/emergency department/hospital, long-term care home/police/fire department/other). The multivariate model predicting the likelihood of psychiatric hospitalization is in Table 4. Predictors were a history of psychiatric hospitalization, suicidal thoughts, and a diagnosis of schizophrenia/schizotypal/delusional disorders or mood disorders.

**Table 4:** Multivariate model predicting psychiatric hospitalization.

Predictors	Odds ratios	*p* value
Age > 75 years old	0.50	0.117
Female	1.50	0.205
Patient/family/friend request for consultation	0.82	0.546
History of psychiatric hospitalization	2.51^***^	0.004
Number of psychotropic treatments	1.21	0.122
MMSE score	0.99	0.834
Level of autonomy	1.83	0.163
Suicidal thoughts	4.40^***^	<0.001
Delusions, hallucinations	1.60	0.347
Somatic complaints	0.61	0.461
Suicidal behavior, self-injurious behavior	2.27	0.240
Seclusion, withdrawal, odd behavior	11.35	0.056
Other	0.22	0.194
ICD-10 psychiatric diagnoses		
F00-F09 organic, including symptomatic, mental disorders	0.86	0.817
F10-19 mental and behavioral disorders due to psychoactive substance use	1.58	0.507
F20-F29 schizophrenia, schizotypal and delusional disorders	8.67^**^	0.004
F30-F39 mood (affective) disorders	2.99^*^	0.049
F40-F48 neurotic, stress-related and somatoform disorders	1.05	0.933
F50-F59 behavioral syndromes associated with physiological disturbances and physical factors	0.00	0.988
F60-F69 disorders of adult personality and behavior	1.27	0.790
Observations	283	
R^2^ Tjur	0.295	
Akaike information criterion	326.906	

*p < 0.05, ** *p* < 0.01, ***p < 0.001.

MMSE, mini mental score examination; ICD-10, international classification of diseases, 10th Revision.

## 4 Discussion

Here we aimed to describe the characteristics of older people consulting for psychiatric emergency services and their care pathways. More than 40% of such patients had a history of at least one psychiatric hospitalization and 44% had consulted a psychiatrist in the previous six months. Psychiatric disorders were mainly mood disorders; neurotic, stress-related and somatoform disorders; and schizophrenic, schizotypal and delusional disorders. For the patients admitted to a psychiatric hospital (39%), predictors were a history of such hospitalization, suicidal thoughts and schizophrenia/schizotypal/delusional disorder or mood disorder diagnoses.

To the best of our knowledge, these are the first data in Europe on older people presenting to psychiatric emergency departments, despite the prevalence of psychiatric disorders and symptoms in old age. We chose the age of 60 years and older to define older patients, as this is the minimum age for admission to a long-term care home in France. Four previous studies chose the same threshold, and five other studies chose a threshold of 65 years or older.

Our older patients were predominantly female, as in most studies (5, 7, 8, 10). Only the two Japanese studies (12, 13) found a majority of men, but this seems to be related to the Japanese psychiatric system, in which patients are often brought in by the police.

For more than half of our patients, the patient, a friend or a member of the patient’s immediate family contacted the CPOA. This finding is consistent with previous publications describing how the patient arrived at the psychiatric emergency department (5–8, 10).

More than 40% of the patients had a history of at least one psychiatric hospitalization, which agrees with the few studies reporting this [22% (13), 36% (6), 39% (7)]. Also, 44% of our sample had consulted a psychiatrist in the 6 months before arriving at the psychiatric emergency department. In another sample (6), 18% of patients were in therapy when they presented to the psychiatric emergency service, and in another study (13), 50% had no history of consulting a psychiatrist. We found that 75% of patients were taking at least one psychotropic medication and 50% were taking at least two. Another study found similar rates: 75% (5).

In our sample, the most common symptoms and reasons for consultation were depression, anxiety, sleep disorders and suicidal thoughts. These results are similar to those found in some studies [depression and anxiety (7, 11)] but not in others [delusions, insomnia, disorientation and hallucinations (6), or violence (12)]. Psychiatric ICD-10 diagnoses in our study were mainly mood disorders; neurotic, stress-related and somatoform disorders; and schizophrenic, schizotypal and delusional disorders. Affective disorders and schizophrenia were common in all studies reporting psychiatric diagnoses (5, 8, 10–13). Only 10% of our sample had a diagnosis of organic, including symptomatic, mental disorders, the lowest rate of any study of older patients in psychiatric emergency service. Rates of dementia or organic brain disease vary widely among studies: 14, 27, 29.9 and 43% of behaviors due to dementia (6, 8, 9, 12); 20% to organic brain disorders (7); 10% to cognitive disorders as primary diagnosis and 15% as comorbid diagnosis (10); 42.9% to F0 (13); 45.5% to organic brain syndrome (5), and 12% to other organic brain syndrome (6). Our low rate of organic disorders can be explained by our psychiatric emergency department not being close to a general emergency department but located in the most famous psychiatric hospital in Paris, so our patient recruitment is quite different (less severe somatic comorbidities, more severe psychiatric comorbidities etc.).

In our study, 39% of older patients were hospitalized, which is close to what was previously reported, despite different health systems: 39% (7), 40% (5), 46% (11), 58% (6), 62% (8). The Japanese studies are not comparable because almost all patients were admitted to hospital after being seen in the psychiatric emergency department (12).

In our multivariate model, the factors predicting psychiatric hospitalization were a history of psychiatric hospitalization, suicidal thoughts and a diagnosis of mood disorder or schizophrenia/schizotypal disorder/delusional disorder. The patients were mainly autonomous, and the mean MMSE score was 24.7 (we could not find a published study to compare with our results). Autonomy and cognitive level did not appear as factors predicting psychiatric hospitalization. Reassuringly, only psychiatric factors influenced the decision for psychiatric hospitalization. Psychiatric hospitalization should never be used as a “stop-gap” solution for patients with loss of autonomy or severe cognitive impairment. Our results can be related to those of a previous study (6) showing that dementia increased the likelihood of hospitalization only if it was associated with a psychiatric diagnosis.

The main strength of our study is that it is the first to be carried out in France and in Europe on older patients attending psychiatric emergencies, which allows for better understanding the needs of this population. Nearly 40% of patients aged 60 years and older required psychiatric hospitalization. In France, psychiatric hospital wards are already saturated. What’s more, the older people often require more help from care teams, and some carers have difficulty coping with the specificities of caring for this type of patient. Hence, we should anticipate (15) and plan for hospital units specifically dedicated (and adapted) to receiving older people, with trained care teams and geriatric psychiatrists (16). However, for older people, a break with their living environment can aggravate a psychological imbalance due to the loss of familiar reference points. The development of mobile psychiatry teams for older people, which is growing rapidly in France (17) and in other countries (18), could allow for considering alternatives to full-time psychiatric hospitalization in certain cases.

The main limitation is that our study is monocentric and the results cannot be generalized because the Paris population, although very large with more than 8 million inhabitants, is essentially urban. Second, many patients refused to participate, which may have biased the results. In France, obtaining patients’ consent to participate in clinical research in emergency departments is difficult. Third, interpretation of the MMSE score in the emergency department must be very cautious. In our department, we routinely perform an MMSE on all patients over 60. Several factors (acute episode, anxiety, stress due to arrival in an emergency department, etc.) can have an impact on the MMSE result, but we take them into account in our interpretation of the MMSE score. Another limitation is that the study did not take into account the level of emergency (urgent/non-urgent or relevant/not relevant) as other studies have done. In our centre, patients are seen according to order of arrival and not level of emergency.

In conclusion, the rapid growth of the older population, combined with the high incidence of mental health problems, will inevitably lead to an increase in the number of consultations of patients aged 60 years and older in psychiatric emergencies (19). Our study highlights the need for further studies of older people in psychiatric emergencies in Europe, to anticipate the needs of this specific population and adapt multidisciplinary mental health care.

## Data availability statement

The raw data supporting the conclusions of this article will be made available by the authors, without undue reservation.

## Ethics statement

The studies involving humans were approved by the Comité de Protection des Personnes EST-1. The studies were conducted in accordance with the local legislation and institutional requirements. The participants provided their written informed consent to participate in this study.

## Author contributions

AP-S: Conceptualization, Investigation, Methodology, Writing – original draft. TG: Conceptualization, Funding acquisition, Methodology, Writing – review & editing. MC: Investigation, Writing – review & editing. JS: Formal analysis, Writing – review & editing. DB: Formal analysis, Methodology, Writing – review & editing. VD-L: Formal analysis, Methodology, Writing – review & editing. JL: Conceptualization, Methodology, Writing – review & editing. FP: Conceptualization, Methodology, Writing – review & editing. MS: Conceptualization, Funding acquisition, Methodology, Writing – review & editing. RG: Conceptualization, Funding acquisition, Methodology, Writing – review & editing.
