# Supplementary material for: Characteristics of older patients in the largest French psychiatric emergency centre
Source: Front Psychiatry. 2023 Dec 12;14:1298497. doi: 10.3389/fpsyt.2023.1298497 (PMC10756208; doi:10.3389/fpsyt.2023.1298497)

Supplementary data / Figure 1: Age of patients by sex

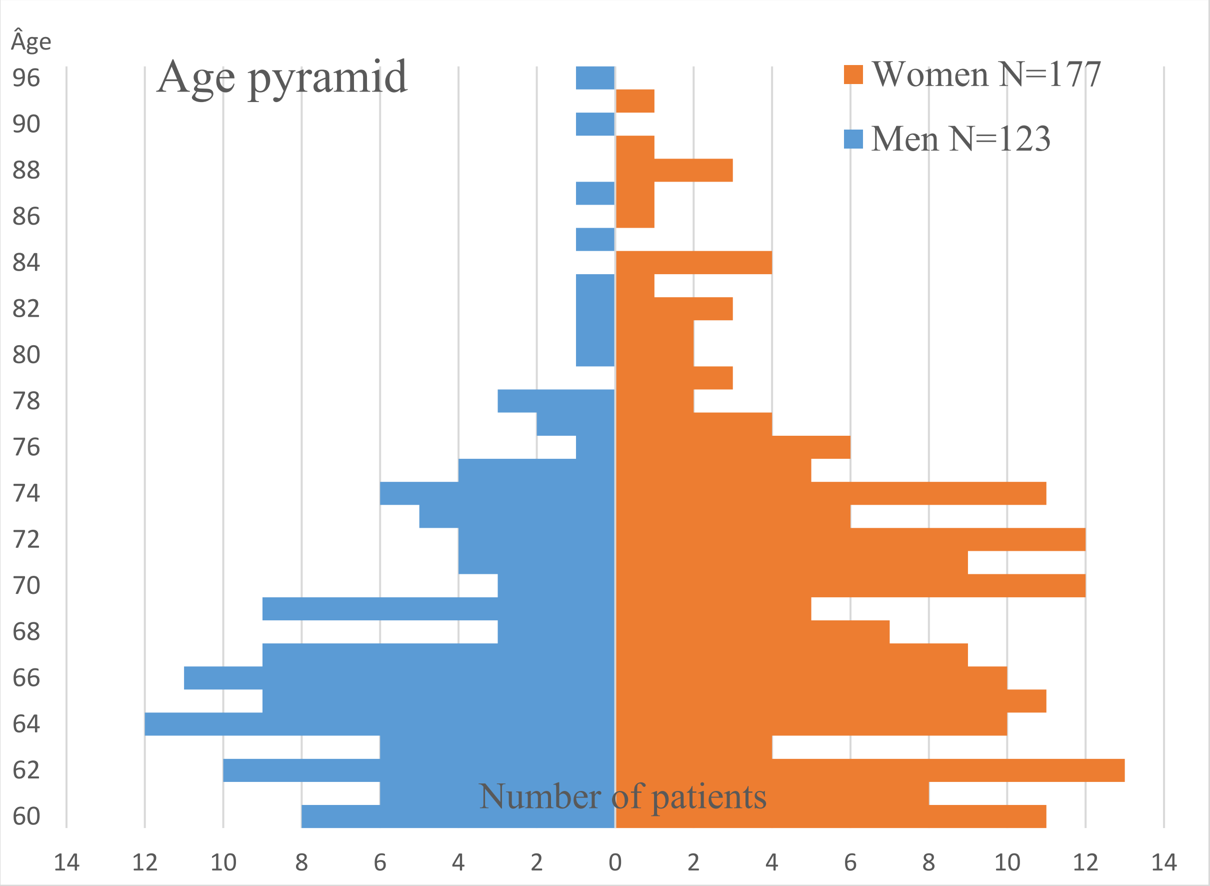

Supplementary data / Figure 2: Number of psychotropic drugs taken

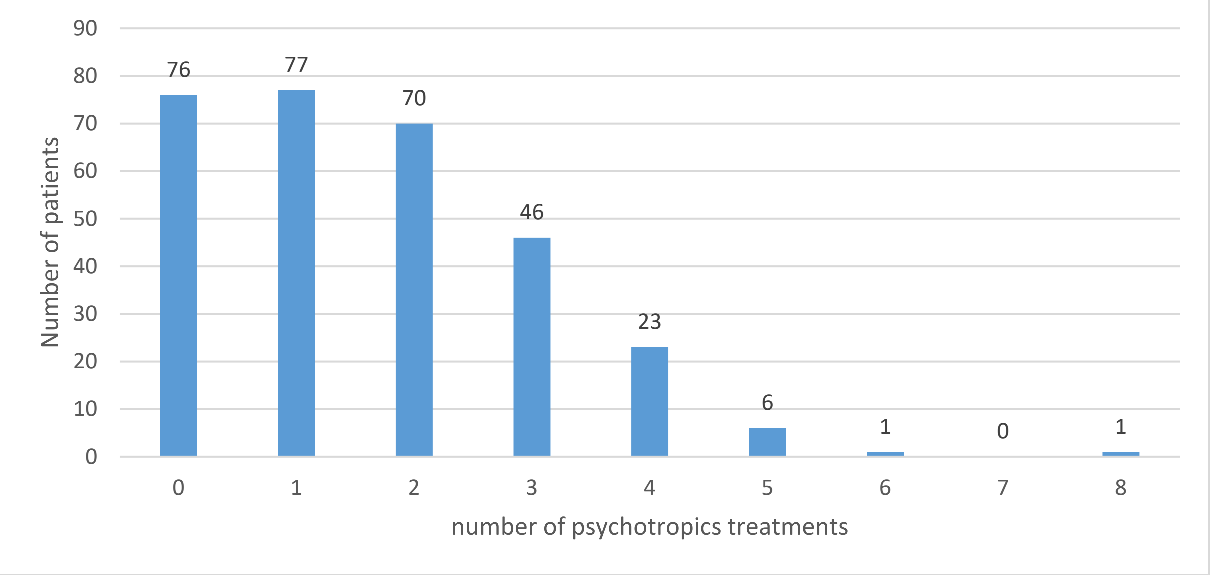

Supplement: Supplementary file 1 [file Data_Sheet_1.pdf]
